# Assessing the factors influencing the quality of pocket-conditioned 3D generative models

**DOI:** 10.1186/s13321-026-01230-5

**Published:** 2026-05-30

**Authors:** Kunyu Wang, Helen Lai, Ross Irwin, Jon Paul Janet, Alessandro Tibo

**Affiliations:** 1 Molecular AI, Discovery Sciences, R&D, AstraZeneca, Gothenburg, Sweden; 2 Molecular AI, Discovery Sciences, R&D, AstraZeneca, Cambridge, UK; 3 Department of Computer Science and Engineering, Chalmers University of Technology and University of Gothenburg, Gothenburg, Sweden

**Keywords:** Flow matching, SBDD, Generative models, 3D generation

## Abstract

Structure-based drug discovery (SBDD) aims to identify novel molecules that bind to therapeutic protein targets. The vast chemical space and limitations of traditional approaches make this task challenging. Recent advances in AI-generative models, such as flow matching, can produce novel, pocket-conditioned molecular structures directly in three-dimensional space. However, most pocket conditioned models in the literature are trained on structures derived from the Protein Data Bank (PDB), which contains structures with varying quality and inconsistent annotation. Moreover, the PDB is enriched with cofactors and natural products, thereby poorly representing real world SBDD scenarios. The relatively limited number of ligand series within the same pockets also hinder the model’s ability to learn protein-ligand interactions effectively. Here for the first time we report the results of training pocket-conditioned generative models on internal crystallography data from a large pharmaceutical company. We also investigate other key determinants of model performance, such as inclusion of hydrogens and pretraining on unconditional data. We evaluate how each factor affects the generative quality of the ligands across the diverse training settings. Our results provide practical guidelines for the development of more effective 3D generative models for SBDD and highlight key directions for future research toward reliable, pocket-aware molecular design.

## Scientific contribution

The paper systematically analyzes the key determinants of performance in pocket-conditioned flow matching models and, for the first time, evaluates training on internal data from a large pharmaceutical company for molecular generation. Our results highlight future directions for developing more reliable and effective 3D generative models.

## Introduction

Structure based drug discovery (SBDD) aims to discover novel molecules that specifically bind target proteins, and plays a critical role at preclinical stage of drug development [3]. Ideally the identified small molecules are expected to form strong interactions with the therapeutic site while remaining high drug-likeness and synthesizability. As the estimated size of chemical space exceeds $10^{63}$ [9], acquisition of qualified molecules is non-trivial. Substantial efforts have focused on developing methods to search for ligands within this near-infinite space. There are traditional approaches such as virtual screening [50], which docks compounds from large commercially-accessible libraries (e.g. Enamine REAL [27] or ZINC [67]) into the target pocket. Most candidates are rejected due to insufficient complementarity, resulting in low absolute hit rates [73]. Another established approach is fragment optimization driven by molecular dynamics and free energy perturbation (MD/FEP) [51], in which fragments are modified with functional groups to lower relative binding free energies. However, calculation for a single protein-ligand complex can take a long time to converge [19], impeding its application to large-scale ligand campaign. In summary, despite successful drug projects have been delivered [70], these techniques generally require substantial computational resource and are suboptimal in efficiency.

Recent advances in artificial intelligence and machine learning methods (AI/ML) have developed generative models as a novel avenue to proactively explore the chemical space. Instead of selecting molecules with desired properties from existing database, molecular generative models can generate novel ligands beyond the training dataset [4, 74]. These models learn the underlying distribution of the chemical space from the training data, enabling the exploration of previously uncharacterized regions. To be applied to a target protein, molecules generated by 2D models must be assigned with appropriate 3D poses by docking protocols. Trained under Reinforcement Learning (RL) frameworks, models can directly generate molecules with improved docking scores [23, 37]. However, these approaches typically require multiple training rounds and costly docking at each round. Since they learn through environment interactions rather than direct pocket conditioning, these methods require thousands of iterations to reach productive performance. Moreover, their accuracy is bounded by the performance of docking protocols themselves. From this perspective, three-dimensional (3D) generative models trained directly on crystallographic data have emerged as especially promising for SBDD [49, 54, 56]. They directly produce atomic coordinates and bond connectivity in 3D, proposing appropriate 3D conformations without docking. Pioneering 3D approaches voxelized atomic densities and employed 3D-CNNs [56], which lacked equivariance and scaled poorly. Later studies represent molecules as 3D graphs and apply equivariant graph neural network (GNN) to address these issues [49, 54]. Many of these methods rely on auro-regressive strategy to generate atoms sequentially [49, 54]. However, because molecular graph lack a natural sequence, similar arguments have been made [34, 40] that stepwise generation imposes an artificial ordering, inherently risking error accumulation or generative bias. As in text and image generation, diffusion [32, 65] and flow-matching [43] architectures offer an alternative one-shot generative way of constructing 3D molecules conditioned on protein pocket [18, 28, 35]. Neural networks are trained either to denoise corrupted 3D molecular graphs (diffusion), or map the objects from known distribution to target data distribution (flow-matching). The time-consuming sampling process is further improved by efficient optimal transport [36].

Although flow-matching models have achieved state-of-the-art performance among pocket-conditioned 3D generative models on general metrics (e.g., validity, uniqueness, and novelty) [18, 36], their application to practical SBDD tasks remains unsatisfactory. Several studies report lower chemical plausibility and drug-likeness in molecules generated by 3D-based models compared with those from public datasets or SMILES-based generative models such as REINVENT [11, 44, 59, 73]. Assessments of generated 3D conformations on different benchmarks also reveal a general suboptimal protein-ligand binding scores before energy-minimization or redocking [6, 44]. Although the casuses are not yet fully understood, several noteworthy factors can be further studied.

First is the extreme sparsity of high-quality training data. Compared to 2D molecule graph databases such as PubChem ($\ge$ 123 million entries [39]), the GEOM-Drugs database contains 37 million 3D conformations generated from only 450,000 molecules [5]. The overall size further shrinks when considering protein-bound information, with around 27,000 experimentally resolved protein-ligand complex structures in the latest PDBBind [47] (v.2024). Many of these structures are not drug like, and the number of ligands per protein (“chemical series") is low, not providing many examples for learning the fine details about protein-ligand interactions needed for SBDD. Quantity is not the only limitation; raw data often contain issues like redundant targets and low-resolution structures, requiring careful cleaning and curation before training. Insufficient experimental data drive scientists resort to computationally modeled data for complementarity. However, whether the unrealistically modeled protein-ligand interactions from redocking introduce new bias into model training remains unknown. There is an urgent call of utilizing protein-ligand complex structure data with enhancement in both quantity and quality for training 3D generative models.

In response to data scarcity, pre-training may increase generative ability. As a standard technique to cope with low data regime, pre-training has improved model performance on other tasks associated with protein-ligand interaction, including molecular conformation generation [77], binding affinity prediction [72] and guided docking [12]. Its benefit for de novo 3D molecule generation is still unclear.

Third, hydrogen atoms strongly influence ligand 3D conformations within the pocket. They determine the formation of hydrogen bonds, which are one of the most common and energetically important mechanisms for specific ligand binding. Despite their crucial role, a lot of models generate only heavy atoms (e.g. C,O,N,S) then use external cheminformatics software (e.g. RDKit) to add them post hoc [34]. The influence of incorporation of explicit hydrogen during training of 3D generative models requires further study.

In this paper, we rigorously investigate the impact of aforementioned factors on pocket-conditioned 3D generative models for de novo ligand generation. Using flow-matching model as the state-of-the-art architecture, we aim to systematically evaluate conditional generative model’s performance across various training settings, providing practical guidelines for effective model development in the field of SBDD. Specifically, we explore the following experimental scenarios:

- Dataset quantity and quality: we analyze the influence of different datasets by comparing models trained on open-source datasets against those additionally trained using a private, high-quality crystallography dataset from industry for training, as well as the impact of combining data sources.
- Effect of pre-training: we study whether pre-training on unconditional molecular data prior to conditioning on protein pockets improves performance.
- Role of hydrogens: we assess the impact of including hydrogen atoms during by comparing models trained with and without explicit hydrogens.

## Related work

3D molecular generation:   Advances in diffusion- and flow-based generative modelling frameworks have recently led to significant strides in 3D molecular generation. The Equivariant Diffusion for Molecules (EDM) model [33] applied a diffusion generative framework [32, 65] to continuous and discrete data in order to jointly sample atom types and 3D coordinates. This approach was later extended [41, 69] to include formal charges and bond orders, and to improve the quality of the generated molecules. Since molecular diffusion models often suffer from very long sampling times, flow matching [2, 43, 46] models such as FlowMol [20] and SemlaFlow [36] have been proposed to reduce generation times.

Pocket-conditioned generation:   In addition to unconditional generation, models which generate ligands within a target protein pocket have also seen a recent surge of interest. These models jointly represent the protein pocket and ligand in 3D space, and learn a ligand generative model, where the protein pocket acts as a condition for the generation. Earlier models, such as 3D-SBDD [49], Pocket2Mol [54], and Graph-BP [45] applied auto-regressive generation within a fixed protein pocket to produce ligand atoms one-by-one. Later approaches, such as DiffSBDD [60], TargetDiff [28] also applied diffusion frameworks to the same setting. Other models have since been proposed which extend the core generative framework. PILOT [17] guides the generation towards ligands with desirable chemical properties, and FlexSBDD [76] and DrugFlow [61] attempt to produce induced-fit effects within the pocket geometry while generating a ligand. Other approaches focused on producing molecules with improved docking scores [22, 55]. More recently, FLOWR [18] proposed to use a separate encoder model for the protein pocket and trained a ligand decoder using flow matching. This led to a significant improvement in the quality of ligand binding pose and reduced sampling time up to 70-fold. Various methods have also been proposed to generate ligands conditioned on desired pharmacophoric interactions with the pocket [1, 18, 42, 78]. There has also been a recent interest in methods to improve the synthesisability of generated molecules by jointly generating ligands along with possible synthesis routes [57, 63].

Ligand evaluation:   Further scrutiny of pocket-conditioned generative models has revealed a number of lurking problems. Many earlier models were found to generate chemically unrealistic molecules – forming no enthalpically-favourable interactions with the protein [31]. They also tended to generate molecules with highly strained binding poses, and were heavily reliant on post-processing minimisation or re-docking [7, 59]. Some more recent models have, however, shown an ability to generate much higher-quality poses [18, 61]. An additional concern is how well generative models are able to generalise to proteins which are underrepresented in structural datasets. Studies on deep-learning docking models have shown that performance drops considerably when models are presented with structurally novel pockets [58, 64]. While some studies have attempted to combat this [18, 21], the lack of diverse protein-ligand structural data means generalisability is likely to remain a problem.

## Methods

### Flow matching for molecular generation

Flow matching (FM) has recently been introduced as a method for learning a mapping from an easy-to-sample prior distribution $p_{noise}$ to a target distribution $p_{data}$, which describes the training data, by following a time-dependent probability path $p_t$. Frameworks for generating continuous data [2, 43, 46], and for discrete data [14, 24] have been proposed, providing powerful techniques for training generative models. Since we wish to generate multi-modal molecular data (continuous atom coordinates and discrete atom and bond types and formal charges) we describe both in more detail below.

Flow matching for continuous data:   We aim to construct a vector field $v_t$ which *generates* a probability path $p_t$ such that $p_0 = p_{noise}$ and $p_1 \approx p_{data}$. Lipman et al. [43] show that this can be achieved by first defining a conditional probability path $p_{t|1}(x_t|x_1)$ and an associated conditional vector field $u_t(.|x_1)$ which *generates* $p_{t|1}(x_t|x_1)$. Crucially,  Lipman et al. [43] show that we can regress $u_t(.|x_1)$ against a learned unconditional vector field $v^{\theta }_t(x_t)$ and that this vector field will generate our desired unconditional probability path $p_t$. At inference time, we generate an initial sample from our prior distribution $x_0 \sim p_0(x_0)$ and then integrate this sample through time, following $v^{\theta }_t$ until $t=1$ to generate a sample from the target distribution [15].

In particular, in this work we define the prior distribution as a unit Gaussian $p_0 = \mathcal {N} (0, I)$ and follow a linear interpolation for molecular coordinates $x_t = tx_1 + (1-t) x_0$ where $x_0 \sim p_0(x_0)$. One vector field which can be shown to generate this path [2, 43] is given by $u_t(x_t | x_1) = \tfrac{x_1 - x_t}{1- t}$. A neural network can then be trained to regress this vector field.

However, rather than learning a vector field, a number of models [36, 41, 69] have found it helpful train networks to directly predict the target $x_1$. This leads to the following loss function for continuous data:

1 $$\begin{aligned} \mathcal {L}_{MSE} = \Vert f_{\theta } (x_t) - x_1 \Vert ^2, \end{aligned}$$

where $f_{\theta }$ is a neural network with parameters $\theta$ that predicts $\hat{x}_1$ from $x_t$, $x_t \sim p_{t|1}(x_t | x_1)$ and $x_1 \sim p_1(x_1)$. In this paper, we follow the same convention and directly learn a denoising model. At inference time we can generate target samples by following the vector field $v_t(x_t) = \frac{{\hat{x}_1 - x_t}}{1-t}$ where ${\hat{x}}_1 = f_{\theta }(x_t)$.

Discrete flow matching:   Various generative frameworks based on continuous-time markov chains have recently been proposed to enable the generation of categorical data [13, 14, 24]. Here, we apply the discrete flow matching framework proposed in Campbell et al. [14]. We denote categorical data by $h_1 \in \{1,...,S\}^D$, where *D* is the number of dimensions and *S* is the number of possible discrete states.

At sampling time, a sequence trajectory $h_t$ transits between different states following probability $p_t$. At each time stamp, $h_t$ moves to the next state *j* with probability determined by the rate matrix $R_t\in \mathbb {R}^{S \times S}$. As for the continuous case, instead of explicitly using $R_t$, we consider the expectation on the conditional rate matrix $R_t(h_t, j|h_1)$ that generates the conditional probability flow $p_{1|t}(h_1|h_t)$:

2 $$\begin{aligned} R_t(h_t, j):= \mathbb {E}_{p_{1|t}(h_1|h_t)}[R_t(h_t, j|h_1)] \end{aligned}$$

$p_{1|t}(h_1|h_t)$ can be approximated by a neural network (parametrized by $\theta$) trained with the cross-entropy loss function:

3 $$\begin{aligned} \mathcal {L}_{CE}(\theta )=\mathbb {E}_{t,h_1,h_t}[\log p_{1|t}^{\theta }(h_1|h_t)] \end{aligned}$$

where $t \sim U[0,1]$, and $h_t$ is sampled from $p_{t|1}(h_t|h_1)$, which linearly interpolates between $p_{t|1}(x_t|x_1)=\frac{1}{S}$ at $t=0$ and $p_{t|1}(x_t|x_1)=\delta \{x_1, x_t\}$ at $t=1$.

### Pocket-conditioned flow-matching model for 3D molecule generation

This work focuses on protein-conditioned molecular generation within the flow-matching framework. We build on SemlaFlow [36], a state-of-the-art flow-matching architecture for unconditional 3D molecule generation, and extend it to incorporate explicit pocket information. SemlaFlow is designed to support both continuous and discrete flows, enabling joint generation of atomic coordinates, atom types, formal charges, and bond orders within a unified framework (see "Flow Matching for Molecular Generation" Section). In the following section, we outline the architectural modifications and conditioning mechanisms required to adapt SemlaFlow for pocket-informed molecular design.

Definition and notations:   Let $\mathcal {X}$ denote the space of all valid molecules, and let $X_1 \in \mathcal {X}$ be a particular molecule. A molecule with *n* atoms is represented as the tuple $X_1 = (x_1, a_1, b_1, c_1)$, where $x_1 \in \mathbb {R}^{n \times 3}$ contains the 3D coordinates of all atoms, $a_1 \in \mathcal {A}^{n}$ contains the atom types, $b_1 \in \mathcal {B}^{n \times n}$ is the adjacency (bond) matrix, and $c_1 \in \mathcal {C}^{n}$ contains the formal charges. The set $\mathcal {A}$ consists of the supported atom types (e.g., H, C, O, N), the set $\mathcal {B} = \{\text {none}, \text {single}, \text {double}, \text {triple}, \text {aromatic}\}$ contains the supported bond types, and the set $\mathcal {C}$ contains the supported formal-charge categories (e.g., $-1$, 0, 1). In this framework, we consider only the protein pockets surrounding the ligand, rather than the entire protein structure, to improve computational efficiency. Protein pockets are defined analogously to molecules; however, unlike molecules, proteins are often provided as PDB files in which explicit bond information is unavailable and must be inferred. Therefore, we simplify the representation by defining the protein pocket solely in terms of atom coordinates and types. We denote the protein pocket space as $\mathcal {Y}$, where each pocket $Y\in \mathcal {Y}$ is represented as a tuple $Y=(x, a)$. The representation is analogous to that of a molecule tuple, except that the here we do not include formal charges.

Architecture adaptation:   We update the SemlaFlow architecture to include $Y\in \mathcal {Y}$ as an input condition for the generative process. Formally, we denote the SemlaFlow architecture with $\hat{X}_1 = f_{\theta }(X_t, Y)$, where $X_t$ is a noisy molecule, *Y* the protein pocket, $\hat{X}_1$ is the predicted molecule from the SemlaFlow, and $\theta$ are the model’s parameters. The model is trained (see [36]) by minimizing the regression term of Eq. 1 and the cross-entropy term in Eq. 3

4 $$\begin{aligned} \mathcal {L}_{SemlaFlow}(\theta ) = \lambda _x \ \textrm{MSE}({\hat{x}}_1, x_1) + \lambda _a \ \textrm{CE} ({\hat{a}}_1, a_1) + \lambda _b \ \textrm{CE} ({\hat{b}}_1, b_1) + \lambda _a \ \textrm{CE} ({\hat{c}}_1, c_1). \end{aligned}$$

The protein’s $n_p$ coordinates are passed through a linear layer that maps them to *d* coordinate sets, producing a tensor $x_p \in \mathbb {R}^{n_p \times 3 \times d}$. The $n_p$ atom types, represented as one-hot vectors, are first embedded and then processed by a simple MLP consisting of a linear layer, a SiLU activation, and a second linear layer, yielding a tensor $h_p \in \mathbb {R}^{n_p \times d}$. The resulting features $x_p$ and $h_p$ are passed to each *SemlaLayer*, which consists of a feature feed-forward layer (see [36], "Flow matching for molecular generation" section) and a graph attention layer (see [36], "Pocket-conditioned Flow-matching model for 3D molecule generation" section). We modify each SemlaLayer by adding a protein-specific feature feed-forward layer and further adapting the graph attention (GA) layer, which is the core modification required to condition SemlaFlow on protein pockets. Each GA layer augments the ligand–ligand message passing with ligand–protein messages. These additional messages are produced by a ligand-protein message module and incorporated into both the invariant and equivariant attention pathways. The protein coordinates and features, $x_p$ and $h_p$, are first normalized using an equivariant map $x_p^{\textrm{norm}} = \phi _{\textrm{equi}}(x_p)$ and an invariant map $h_p^{\textrm{norm}} = \phi _{\textrm{inv}}(h_p)$, respectively. Then, for every ligand–protein atom pair (*i*, *j*), the model constructs invariant messages by concatenating ligand and protein features,

$$\mu ^{\textrm{inv}}_{i,j} = \bigl [h^{\textrm{norm}}_i \,\Vert \, h_{p,j}^{\textrm{norm}}\bigr ],$$

and equivariant messages based on the Euclidean distance between normalized ligand and protein coordinates (*i*, *j*) across all coordinate sets *s*,

$$\mu _{i,j,s}^{\textrm{equi}} = \sqrt{\bigl \Vert x^{\textrm{norm}}_{i,s} - x^{\textrm{norm}}_{p,j,s}\bigr \Vert ^2 + \varepsilon }.$$

**Fig. 1:**
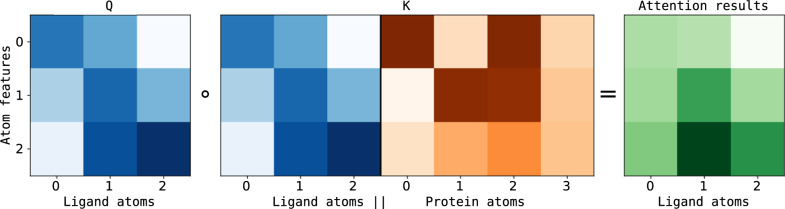
Illustration of the attention operation between ligand and protein. The symbol $\circ$ denotes attention between the left (*Q*) and middle (*K*) matrices, computed as $\text {softmax}(QK^T)K$. For simplicity, we assume a ligand with 3 atoms and a protein with 4 atoms, each with feature dimension 3. The left matrix contains ligand features. The middle matrix contains the concatenated ligand–protein atom features, forming a $3\times 7$ matrix. The right matrix shows the resulting attention output between the ligand (*Q*) and the concatenated features (*K*)

These messages of sizes $\mathbb {R}^{n\times n_p\times d}$ and $\mathbb {R}^{n\times n_p\times d}$, respectively, are then concatenated to the corresponding invariant and equivariant messages $m^{\textrm{equi}}$ and $m^{\textrm{inv}}$ applied to the ligand invariant and equivariant features (see [36] "Pocket-conditioned Flow-matching model for 3D molecule generation" section). Ultimately, only the ligand nodes are updated, ensuring that the protein acts purely as a conditioning context while preserving the ligand-centric nature of the model. Figure 1 illustrates how the protein conditioning attention mechanism works.

## Experiments

### Datasets

The pre-training of unconditional SemlaFlow model is performed on GEOM-Drugs [5] dataset, which consists of 317,928 high-quality simulated conformers. Two protein-ligand complex datasets were used for training and testing conditional models in the paper. The first one is PDBBind [71] (v.2020), a public benchmark dataset containing 19,443 complex structures collected from experimental measurements. The other dataset comprises of a 9000-structure subset taken from Resolve, AstraZeneca’s in-house X-ray crystallography database of high-resolution protein-ligand complexes. Resolve is maintained and provided by Discovery Sciences, R&D, AstraZeneca.

Before application, both datasets were processed with the same cleaning steps using the Schrödinger suite [62]. The protein in each complex is screened based on their distances to the ligand, so that only the chains closest to ligand are kept. The processed complexes are then prepared with Schrödinger’s PrepWizard for geometry correction and protonation states assignment. Successfully processed protein-ligand complexes are then evaluated the docking scores within the binding pocket by Glide [30]. Only complexes with negative Glide scores were retained, giving a final set of 18,990 PDBBind training samples. Binding pockets are extracted out of the remaining complexes following the similar settings in  Zhang et al. [75]. Specifically, any residue that has over 10 atoms (including hydrogens) and has at least one atom within 3.5Å of the native bound ligand, will be included in the binding pockets. These selected binding pockets and their bound ligands form the datasets for next step of training.

We constructed the test set from PDBBind via time-based splitting following DiffDock [16]. Any training complex whose ligand overlapped with the test set was excluded. Test complexes were processed with the same pipeline as for the training data. The final testset comprises 120 protein–ligand complexes.

The pocket-conditioned SemlaFlow is also trained and tested on SPINDER dataset [18], which is the largest high-quality filtered subset of PLINDER (released 06/2024) [21]. SPINDER incorporates extensive structural refinement while perfectly preserves the same train-test splits of the original PLINDER dataset.

### Training and testing

Models without pretraining were trained on their corresponding protein-ligand complex dataset using the Adam optimizer for 300 epochs with batch size of 2.

The pretrained model was trained on the GEOM-Drugs database using the Adam optimizer for 30 epochs. It was then fine-tuned on PDBBind and Resolve for 300 epochs. During fine-tuning, random batches of two molecules from the GEOM-Drugs dataset were interleaved with protein–ligand pairs in an unconditional setting to prevent the model from forgetting general chemistry, as the protein–ligand datasets together contain fewer than 20,000 complexes.

Models trained under different set-ups are all tested with datasets generated from the same receptors and procedures. For each of 120 protein pockets in the testing set, 128 ligands was generated. These combined datasets are used for evaluations of benchmark scores. To assess the actual 3D generative ability, generated ligands from the models are directly used for testing without re-docking.

Models trained on SPINDER followed the same process as models without pretraining.

### Evaluation metrics

To compare model ability in target-conditioned generation fairly, we did comprehensive evaluation for generated molecules under a series of metrics across various aspects, including chemical plausibility, 3D conformation, and binding mode. Valid rate is computed as the fraction of valid molecules over all generated molecules, where “valid" is defined as (1) being constructed by “SDMolSupplier" function in RDKit (v2024.09), and (2) being successfully sanitized by RDKit(v2024.09) using function “Chem.SanitizeMol".

**QED** is the average Quantitative Estimation of Drug-likeness score [8] over all valid generated molecules. QED combines a couple of physicochemical and structural properties to estimate the drug-likeness of molecule.

**PBR** (PoseBusters Ratio) represents the proportion of molecules that passed all 18 tests in PoseBusters test suite [11]. PoseBusters test suite is a collection of 18 metrics designed to identify chemically-inconsistent or physically-implausible binding pose in docking.

**Strain Energy** measures the energy change of the generated pose before and after the energy minimization in MMFF94 force field without pocket constraint. Generated molecules are first constructed by “SDMolSupplier" then optimized with “MMFFOptimizeMolecule" function in RDKit with maximum 200 iterations. It’s an assessment of whether the particular 3D conformation adopted by the molecule is energetically-favorable.

Docking-based estimation of binding enthalpy between generated ligand and target protein is assessed by score functions of two docking protocols, AutoDock Vina and Glide. Molecules are evaluated using the generated poses in situ and after local energy minimization, without re-docking.

**VS** represents the average AutoDock Vina score of all valid generated molecules binding to their target pocket.

**GS** represents the average Glide score of all valid generated molecules binding to their target pocket.

**BNR** (Better than Native Ratio) is, for each protein system, the proportion of generated, valid molecules whose Glide scores are lower than that system’s native bound ligand, and the reported BNR is the average of this proportion across protein systems.

**#HB.** counts the hydrogen bonds formed between generated ligand and pocket residues, detected by ProLIF [10].

**Int.R** is the average of the proportion of ligand-protein interactions formed by generated ligands that match the native interactions.

**Solved.R** represents the proportion of generated structures for which AiZynthFinder [25] identified at least one synthetic route. As only structures with negative docking scores are typically considered viable candidates, and synthesis planning is computationally expensive, this metric is computed only for structures with negative docking scores. In this setup, AiZynthFinder uses a public compound stock for synthesis planning, while the template model is trained on internal data, providing improved performance and broader coverage of reaction space.

### Chemical space illustration

We plot the 2D coverage of generated molecules in chemical space with UMAP [52]. Constructed molecules are sanitized and computed by RDKit to acquire their Morgan fingerprint vectors with 2048 bits. The high-dimensional vectors are then transformed into 2D representations via UMAP. A baseline subset is constructed by randomly selecting 2% of GEOM-Drugs database uniformly across atom counts 4–60. The UMAP transformation computed from this subset is used for all dimensional reductions of data distribution plotted with UMAP.

### Local energy minimization

Molecules are optimized by minimizing according to the MMFF94 force field, considering the local impact of pocket. We extended the two non-bonded terms of MMFF94, van der Waals and electrostatic interaction, to account for the interactions between ligand and target protein used to condition the generations. This extended MMFF94 energy is formally defined as:

5 $$\begin{aligned} E(X,Y) = \text {MMFF94}(X) + E_{vdW}(X,Y) +E_{Q}(X,Y), \end{aligned}$$

where $E_{vdW}(X,Y)$ and $E_{Q}(X,Y)$ denote the Van der Waals and electrostatic interactions between a ligand $X$ and a protein $Y$. The implementation is available at https://github.com/MolecularAI/TorchMMFF94.

## Results and discussion

### Effect of different datasets

In this section, we investigate the impact of different training data on model performance. Generative models with the same architectures and settings are trained with three types of dataset compositions: only PDBBind, only Resolve, both PDBBind and Resolve (abbreviated as PDBBind model, Resolve model and “both" model hereafter). Before trained on complex dataset, all models were first pre-trained unconditionally on GEOM-Drugs. All models generate explicit hydrogen atoms. Scores of generated ligands have been measured without re-docking, but we also provided scores after local energy minimization within the pocket for comparing.

**Fig. 2:**
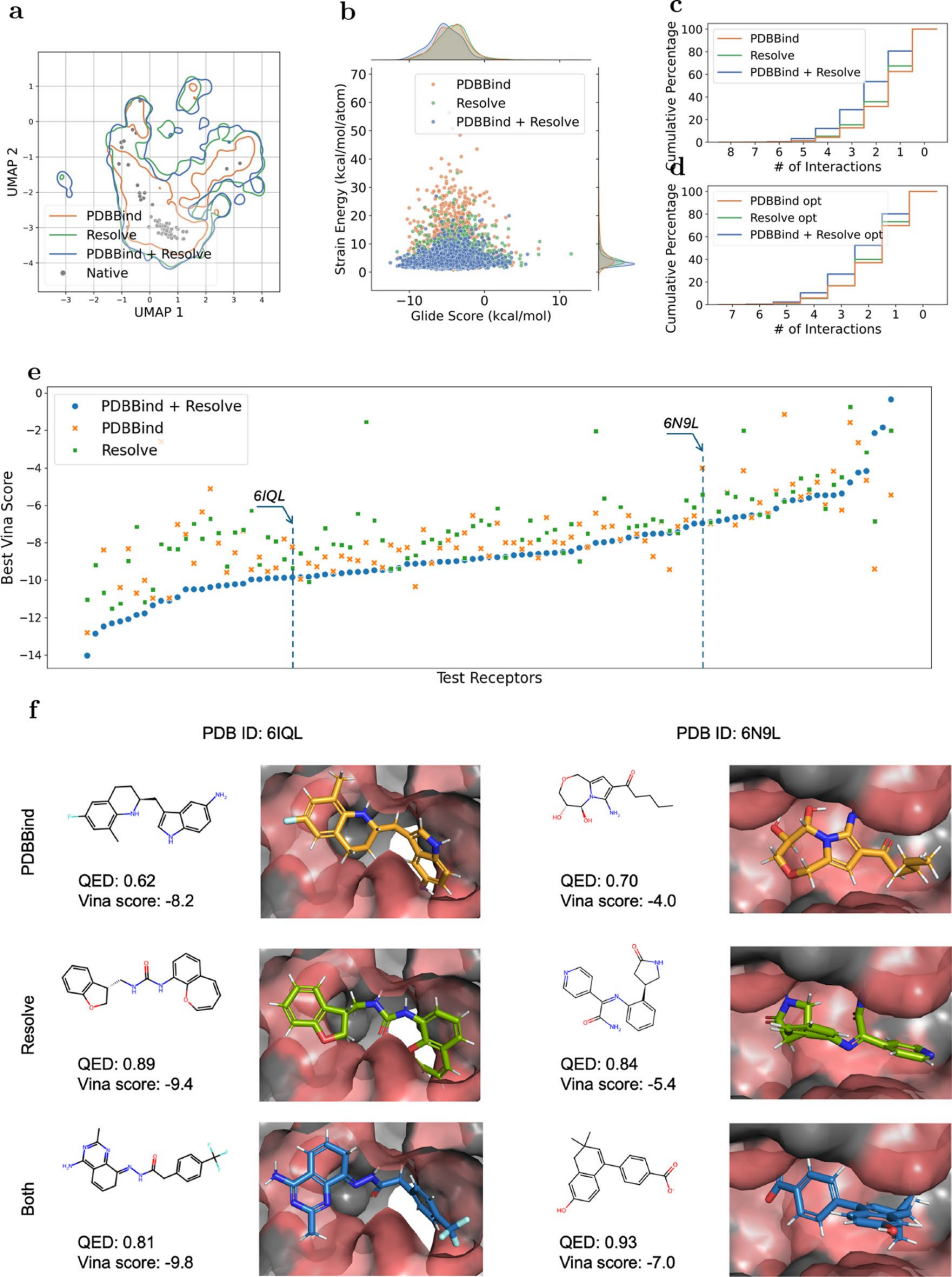
Comparison of results on the PBDBind test set for models trained on different datasets. **a** UMAP of sampled ligands **b** Ligand dot plot by Glide score (<10,000 kcal/mol) and strain energy before local optimization. **c**, **d** Number of protein-ligand hydrogen bonds before and after local optimization. **e** Best vina scores obtained for each test case. **f** Visualization of some sampled poses for three test systems, demonstrated molecules are after optimization

We assess the 2D molecular graph quality of generated molecules in terms of validity, drug-likeness and chemical space coverage. Although containing only half of the number of training molecules, the Resolve model generated more valid molecules (75.62%) compared to PDBBind model (69.55%) (see Table 1). The generation quality is further improved for “both" model, which generated 10% more valid molecules compared to Resolve model (see Table 1). The drug-likeness of molecules is represented as the average QED scores of valid generated molecules. In evaluations for all three types of models, Resolve model and “both" model achieved similar QED (0.62 and 0.63), while PDBBind model have a slightly higher average QED (0.66) (see Table 1). Chemical spaces learned by three models all stay close proximity to the distribution of native ligands in test dataset. Notably, models that include Resolve in the training data demonstrated a broader chemical space coverage (see Fig. 2a), indicating the stronger capacity to generative diversity.

**Table 1:** Molecule quality metrics for the SemlaFlow model

Method	QED	PBR (%)	BNR (%)	Valid (%)	# HB	S. Energy	Int.R (%)	Solved.R (%)
PDBBind	$0.66 \pm 0.16$	27.8	$2.9 \pm 8.7$	69.55	$1.05 \pm 1.13$	$6.58 \pm 8.48$	$49.4 \pm 18.8$	34.67
PDBBind + Opt	$0.66 \pm 0.16$	60.5	$6.9 \pm 15.6$	69.55	$1.25 \pm 1.19$	$1.04 \pm 2.42$	$49.7 \pm 18.2$	34.67
Resolve	$0.62 \pm 0.20$	31.8	$2.8 \pm 9.6$	75.62	$1.18 \pm 1.16$	$4.88 \pm 3.19$	$47.4 \pm 19.6$	32.12
Resolve + Opt	$0.62 \pm 0.20$	64.7	$6.6 \pm 17.1$	75.62	$1.30 \pm 1.15$	$1.09 \pm 2.11$	$47.6 \pm 17.9$	32.12
PDBBind + Resolve	$0.63 \pm 0.20$	46.0	$5.9 \pm 14.8$	86.20	$1.69 \pm 1.32$	$3.65 \pm 1.96$	$55.3 \pm 17.5$	32.24
PDBBind + Resolve + Opt	$0.63 \pm 0.20$	76.0	$8.8 \pm 19.0$	86.20	$1.64 \pm 1.29$	$0.98 \pm 2.07$	$52.2 \pm 17.6$	32.24

*QED* quantitative estimate of drug-likeness, *PBR* PoseBuster pass ratio, *BNR* better-than-native ratio, *Valid* validity ratio, *S. Energy* strain energy, *# HB.* hydrogen bond count, *Int.R* interaction recovery ratio, *Solved.R* AiZynthFinder solved ratio

Next we assessed the 3D molecular structural quality in different aspects. Higher strain energy indicates existing unrealistic bond lengths or torsion angles. The strain energy in descending order is 6.58 kcal/mol/atom for PDBBind model, 4.88 kcal/mol/atom for Resolve model, and 3.65 kcal/mol/atom for “both" model (see Table 1). PoseBuster (PB) is a more comprehensive test suite composed of 18 metrics involving both 2D graphs and 3D conformations. PBR is the fraction of valid molecules that passes all testing metrics. PBR for PDBBind model, Resolve model, “both" model is 27.8%, 31.8%, 46.0%. The model trained with both databases, also the largest training data, has the highest PBR of 46.0% (see Table 1), indicating a general superior quality. Overall, addition of Resolve data into training data helps in generating more geometrically valid and physically realistic molecules. After local energy minimization, strain energies of molecules from all three models were significantly reduced to around 1.0 kcal/mol/atom (see Table 1). In summary, model trained with larger dataset and more high-quality data has the best performance across 2D and 3D quality metrics.

**Table 2:** Docking score evaluation for the SemlaFlow model

Method	$\text {VR} < 0$ (%)	$\text {GR} < 0$ (%)	VS	GS
PDBBind	47.00	19.41	$3.04 \pm 16.43$	$-4.45 \pm 2.08$
PDBBind + Opt	64.98	53.85	$-4.23 \pm 7.45$	$-5.21 \pm 2.06$
Resolve	48.40	23.68	$3.98 \pm 18.28$	$-4.19 \pm 2.17$
Resolve + Opt	69.26	60.24	$-3.70 \pm 10.44$	$-4.97 \pm 2.25$
PDBBind + Resolve	67.20	38.82	$0.32 \pm 15.66$	$-5.16 \pm 2.17$
PDBBind + Resolve + Opt	80.12	71.88	$-4.51 \pm 8.22$	$-5.59 \pm 2.20$

* VR < 0* negative vina score ratio, *GR < 0* negative docking score ratio; *VS* vina score, *GS* glide score (mean docking score)

We next analyze various surrogates that assesses the target binding affinity of generated molecules. Here we choose AutoDock Vina [68] score and Glide score [30] as two main metrics, which are both commonly used docking scores. The “both" model presents a significant advantage over the other two models in terms of both metrics, with over 67% molecules having negative Vina scores and over 38% having negative Glide scores (see Table 2). Compared to the other two models, the average Vina score and average Glide score are the lowest as well. Such relative advantage is preserved even after local energy minimization. Although all three sets of molecules show improved binding scores after relaxation, molecules generated by the “both" model make the greatest progress, with over 80% ligands having negative Vina scores and over 70% ligands have negative Glide scores (see Table  2). The statistics are supported by the Vina score evaluation for single targets across the test set. Figure 2e displays the best Vina scores each model can achieve, selected from the 128 ligands sampled for each of the 120 target proteins. The “both" model is able to generate ligands with the highest binding affinity on over 90% of the receptors. Figure 2f visualizes 3D molecular conformations of the best ligands generated by each of the three models. Ligands with lower Vina scores show a higher pocket occupancy and fewer steric clashes. Analysis of the Glide score - strain energy collective distribution of generated molecules is shown in Fig. 2b. In area where ligand affinities are higher (Glide score $\le$ −5 kcal/mol), level of strain energy is remained generally under 10 kcal/mol/atom, especially for those ligands generated by the “both" model. This indicates that the model can generate better suited poses without the sacrifice of strained geometries. Count of realized hydrogen bonds between ligands and pocket provides another angle to compare different model capabilities in generating realistic binding poses. Over 80% of molecules generated by the “both" model builds at least 1 hydrogen bond with the receptor, while the fractions for the other two models are around 60% (see Fig. 2c). The discrepancy decreased after the local energy minimization (see Fig. 2d).

We also assessed the synthesizability of the generated structures using AiZynthFinder (see "Evaluation metrics" section for detailed settings) across all three settings. Among structures with negative docking scores, approximately 32% of those generated using models trained on PDBBind Resolve combined and Resolve alone were found to have at least one feasible synthetic route. For models trained on PDBBind alone, this proportion was slightly higher, at around 35%. We hypothesize that this difference arises because AiZynthFinder relies on a public compound stock to determine whether a route is solvable. As a result, structures generated from models trained on public datasets may be more aligned with the available stock, making it easier to identify viable synthesis routes. Overall, however, we do not observe a significant difference in synthesizability across the three settings. Given that the models were not explicitly trained to optimize for synthesizability, the proportion of solved molecules is considered acceptable.

Finally, we also experimented with using Boltz-2 [53] to co-fold the six generated ligands in Fig. 2f with their targets to compare the resulting quality of the geometries obtained. In what we believe is the first comparison of the pose quality of pocket-conditioned flow matching models and large co-folding methods, we determined that the overall quality of the poses generated by our method and Boltz-2 are very similar, although they solve very different problems (ligand generation vs protein-ligand pose prediction, see Appendix F).

### Effect of pre-training

After analyzing the impacts of different complex datasets, we then investigate whether pretraining on unconditional ligand datasets could help improve model performance. Two models were trained with different manners: one is trained from scratch solely on PDBBind as the protein-ligand dataset; the other one is first pretrained on GEOM-Drugs [5] dataset for unconditional generative tasks, then fine-tuned on PDBBind for pocket-conditional generation. Explicit hydrogens have been introduced into the generation process for both models. We use the same test subset as in "Effect of different datasets" section, and provide the additional evaluation results after local energy minimization.

**Fig. 3:**
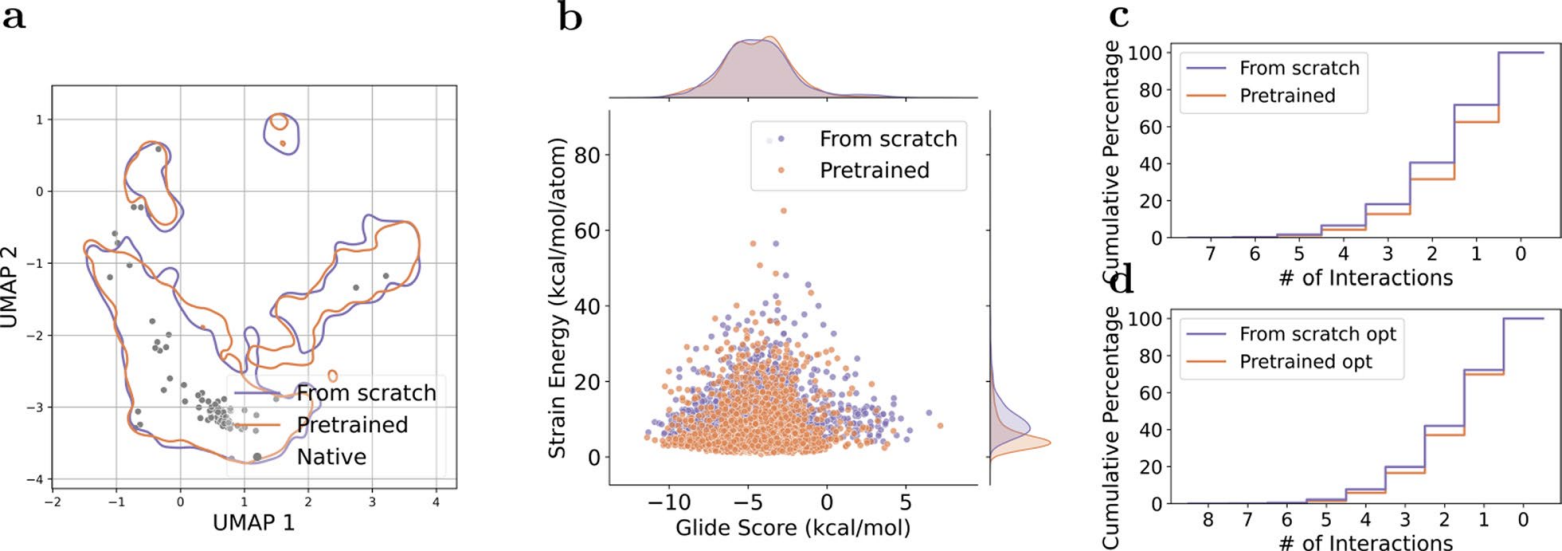
Comparison of results on the PBDBind test set for models trained with and without pretraining. **a** UMAP of sampled ligands **b** Ligand dot plot by Glide score (<10,000 kcal/mol) and strain energy before local optimization. **c**, **d** Number of protein-ligand hydrogen bonds before and after local optimization

In general, there is little difference in the 2D graph quality for models trained under two approaches. Chemical spaces drawn from the generated molecules of two models are highly overlapping, suggesting pretraining has minor impact on generation diversity (see Fig. 3a). The pretrained model can generate slightly more valid molecules (69.55%) compared to the from scratch model (66.11%) (see Table 3). Their generated molecules have shown similar drug-likeness as well (see Table 3). However, pretraining can lead to differentiated performance in terms of 3D conformation quality. The average strain energy value for molecules generated by from scratch model is significantly higher than molecules from pretrained model (10.59 vs. 6.58 kcal/mol/atom) (see Table 3). The overall distribution analysis then demonstrates that it is a global phenomenon for all successfully docked molecules (see Fig. 3b). As a more comprehensive molecule quality checking tool, PBR assessment also suggests a better generative quality of model with pretraining. 27.8% molecules generated by pretrained model passed all the PB testing metrics, compared to only 22.3% molecules for from scratch model (see Table 3). Although local energy minimization substantially improves the strain energy for from scratch model, the average value (1.58 kcal/mol/atom) is still 50% worse than pretrained model (1.04 kcal/mol/atom) (see Table 3). PBR of both models are improved under optimization, while the pretrained model still has the higher ratio (see Table 3). Hence, pretraining on unconditional data before conditional training is beneficial for models to preserve good 3D structure quality.

**Table 3:** Molecule quality metrics for the SemlaFlow model

Method	QED	PBR (%)	BNR (%)	Valid (%)	# HB	S. Energy	Int.R (%)
From Scratch	$0.67 \pm 0.15$	22.3	$4.2 \pm 11.2$	66.11	$1.35 \pm 1.23$	$10.59 \pm 5.92$	$49.8 \pm 19.0$
From Scratch + Opt	$0.66 \pm 0.16$	53.7	$8.7 \pm 19.2$	66.12	$1.45 \pm 1.29$	$1.58 \pm 2.45$	$51.1 \pm 18.1$
Pretrained	$0.66 \pm 0.16$	27.8	$2.9 \pm 8.7$	69.55	$1.05 \pm 1.13$	$6.58 \pm 8.48$	$49.4 \pm 18.8$
Pretrained + Opt	$0.66 \pm 0.16$	60.5	$6.9 \pm 15.6$	69.55	$1.25 \pm 1.19$	$1.04 \pm 2.42$	$49.7 \pm 18.2$

*QED* quantitative estimate of drug-likeness, *PBR* PoseBuster pass ratio, *BNR* better-than-native ratio, *Valid* validity ratio, *S. Energy* strain energy, *# HB.* hydrogen bond count, *Int.R* interaction recovery ratio

**Table 4:** Docking score evaluation for the SemlaFlow model

Method	$\text {VR} < 0$ (%)	$\text {GR} < 0$ (%)	VS	GS
From Scratch	44.81	18.78	$4.20 \pm 18.67$	$-4.39 \pm 2.26$
From Scratch + Opt	82.35	56.53	$-3.40 \pm 10.09$	$-4.78 \pm 2.29$
Pretrained	47.00	19.41	$3.04 \pm 16.43$	$-4.45 \pm 2.08$
Pretrained + Opt	64.98	53.85	$-4.23 \pm 7.45$	$-5.21 \pm 2.06$

*VR < 0* negative vina score ratio, *GR < 0* negative docking score ratio, *VS* vina score, *GS* glide score (mean docking score)

Analysis on ligand binding affinity surrogates shows a different picture. The negative Vina score ratio is 44.81% for model trained from scratch, and 47.00% for model with pretraining, with an average Vina score 4.20 kcal/mol for model trained from scratch and 3.04 kcal/mol for model with pretraining (see Table 4). The pretrained model has a minor advantage, yet this becomes less obvious after the local energy minimization. Although from scratch model obtains a mean Vina score is −3.40 kcal/mol, which is still worse than pretrained model (−4.23 kcal/mol), its negative ratio increases to 82.35%, better than that of pretrained model (64.98%)(see Table 4). Similar trends can also be observed in Glide score statistics. Interestingly, these results are also supported by the assessments of BNR (see Table 3) and hydrogen bonds (see Fig. 3c, d). For either before or after the relaxation, the from scratch model generated ligands have more hydrogen bonds within the pocket, and more of them have better binding scores over native ligands. Models trained with only protein-ligand complex data might focus more on interactions with pocket, while focusing less on the rules of intramolecular geometry, thus rely more on other postprocessing tools i.e. force field to correct invalid structures.

### Effect of incorporating hydrogen atoms during training

In this section, we performed experiments to investigate whether generating hydrogen atoms explicitly would help generation quality. We trained two models using the same model architecture but in one case we retained explicit hydrogen atoms and in the other case committed them. Both models are trained from scratch using only PDBBind dataset without pre-training. One model (“No Hs") generates only the heavy atoms of molecules, whose hydrogen count and positions are defined post-generated by RDKit. These added hydrogens are optimized exclusively under the extended MMFF94 force field ("Local energy minimization" section) for correct assignment. The other model (“Hs") generates molecules with all atoms, including the exact atomic coordinates and positions of hydrogen atoms. No extra optimization is performed. In cases designated for local energy minimization (“No Hs Opt" and “Hs Opt"), a full-atom optimization is performed to the ligand.

**Fig. 4:**
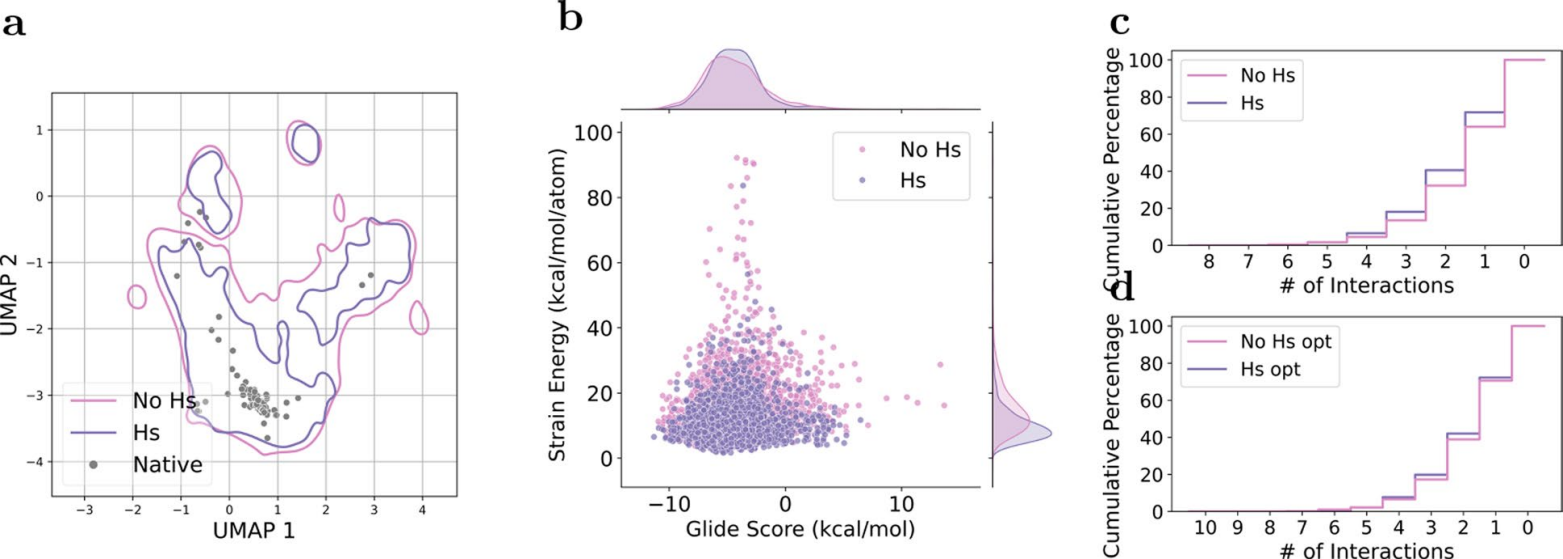
Comparison of results on the PBDBind test set for models trained with and without explicit hydrogens. **a** UMAP of sampled ligands **b** Ligand dot plot by Glide score (<10,000 kcal/mol) and strain energy before local optimization. **c**, **d** Number of protein-ligand hydrogen bonds before and after local optimization

Exclusion of explicit hydrogens has a negative impact on chemical and physical validity of the generated molecules. The fraction of valid molecular graphs is 58.34% for “No Hs" model and 66.11% for “Hs" model (see Table 5). Assessment of PBR again proves the capability of “Hs" model in generating more chemically realistic molecules. PBR of “No Hs" model is less than half of the “Hs" model (10.2% vs. 22.3%), and 4% lower after relaxation (49.3% vs. 53.7%)(see Table 5). Generating explicit hydrogens also helps to improve the physical plausibility of the 3D poses. The “Hs" model produced molecules with an average strain energy of 10.59 kcal/mol/atom before local energy minimization, while “No Hs" model produced molecules with 15.96 kcal/mol/atom (see Table 5). However, from the chemical space coverage perspective, exclusion of hydrogens from training contributes to larger diversity of molecular graphs (see Fig. 4a).

**Table 5:** Molecule quality metrics for the SemlaFlow model

Method	QED	PBR (%)	BNR (%)	Valid (%)	# HB	S. Energy	Int.R (%)
No Hs	$0.68 \pm 0.15$	10.2	$5.3 \pm 14.5$	58.34	$1.13 \pm 1.19$	$15.96 \pm 17.65$	$50.5 \pm 18.6$
No Hs + Opt	$0.68 \pm 0.15$	49.3	$8.2 \pm 19.8$	58.34	$1.33 \pm 1.28$	$1.72 \pm 15.56$	$53.6 \pm 17.8$
Hs	$0.67 \pm 0.15$	22.3	$4.2 \pm 11.2$	66.11	$1.35 \pm 1.23$	$10.59 \pm 5.92$	$49.8 \pm 19.0$
Hs + Opt	$0.66 \pm 0.16$	53.7	$8.7 \pm 19.2$	66.12	$1.45 \pm 1.29$	$1.58 \pm 2.45$	$51.1 \pm 18.1$

*QED* quantitative estimate of drug-likeness, *PBR* PoseBuster pass ratio, *BNR* better-than-native ratio, *Valid* validity ratio, *S. Energy* strain energy, *# HB.* hydrogen bond count, *Int.R* interaction recovery ratio

The impact the on docking scores obtained is less prominent compared to the other metrics. Although evaluation of Vina score on “Hs" model shows a worse average value, its fraction of negative scores is higher (see Table 6). Such an advantage was amplified after local energy minimization, with 82.35% for “Hs + Opt" model and 54.81% for “No Hs + Opt" model (see Table 6). Two models are comparable in terms of average Glide score. Molecules from the “Hs" model have lower negative Glide score ratio and lower BNR ratio, while the results are swapped after local relaxation (see Tables 5, 6, Fig. 4b). It is well noted that compared to Vina score, Glide scores are presumed to be more accurate in evaluating protein-ligand binding, see for example Guo et al. [29]. Therefore, for molecules that can pass the validity check, their binding scores are barely effected by whether explicit hydrogen are generated by the model or added in post-hoc.

**Table 6:** Docking score evaluation for the SemlaFlow model

Method	$\text {VR} < 0$ (%)	$\text {GR} < 0$ (%)	VS	GS
No Hs	41.97	21.89	$2.53 \pm 17.50$	$-4.40 \pm 2.70$
No Hs + Opt	54.81	44.27	$-4.24 \pm 9.16$	$-4.72 \pm 2.63$
Hs	44.81	18.78	$4.20 \pm 18.67$	$-4.39 \pm 2.26$
Hs + Opt	82.35	56.53	$-3.40 \pm 10.09$	$-4.78 \pm 2.29$

*VR < 0* negative vina score ratio, *GR < 0* negative docking score ratio, *VS* vina score, *GS* glide score (mean docking score)

Not surprisingly, there is considerable difference between two models regarding the formation of hydrogen bonds. The identified hydrogen bond count per molecule for the “No Hs" and “Hs" models are 1.13 and 1.35 on average respectively (see Table 5). After local energy minimization, the percentages of hydrogen bond counts are more similar (see Fig. 4d), though the average count per molecule for “Hs" model is still higher. Under our pocket-conditioned generation settings, including explicit hydrogen during training enables model to characterize more hydrogen bond interactions between generated ligands and protein. Cremer et al. [18] found adding explicit Hs slightly reduced the mean interaction recovery rate across test sets. The statistical discrepancy is likely because they only considered ligand hydrogens and they split hydrogen bonds into two categories (“HBDonor" vs. “HBAcceptor"). Our study retained protein hydrogens, thus treating both interaction directions equivalently as hydrogen bonds.

## Conclusion

We conducted a number of experiments to test the performance of a pocket-conditioned SemlaFlow model along three axes: dataset composition; pretraining on unconditional data; and explicitly generating hydrogen atoms. In terms of the data used to train pocket-conditioned 3D generative models, we observed that (1) use of a subset of internal, high-quality drug-like crystal structures resulted in a better model than using the publicly-available PDBBind set, despite the later being more than twice as size; (2) the combination of the internal and publicly-available data resulted in the strongest model, showing clearly that the different sets are complementary. We hypothesize that the internal subset contains more “chemical series" data that helps the model make more accurate fine-grained predictions of binding poses, but that the PDBBind set is beneficial for having more data overall and better coverage of protein space.

Pre-training on ligand-only databases appears to benefit models in generating more rational 3D ligand conformations, but do not materially impact the predictions of protein-ligand complementarity.

Using the generative model to explicitly predict hydrogen atoms significantly increased the graph validity of generated molecules and improved the ability of the model to predict accurate protein-ligand interactions, in contrast to previous work.

This work assessed several variants of generative models for 3D molecular design that we have implemented industrially, and offers practical advice on future training of improved models. Our findings highlight the value proposition of relatively modest sets of high-quality data. One of the main advantages of 3D generative models over 2D models is that they can produce novel ligands (optionally conditioned on some features) directly in the 3D space, enabling us to leverage spatial information (including the protein) explicitly and in a zero-shot fashion. In contrast, 2D generative models typically need to be paired with reinforcement learning (RL) and rely on expensive, large blind searches combined with docking scoring function to identify promising candidates. On the other hand, 2D generative models are typically easier to direct with multiple scoring functions, which means that they cannot yet be substituted by 3D generators. While access to more high-quality data is always important for teaching the model better chemistry and mitigating overfitting, future extensions should consider the impact of adding synthetic data (i.e. docked or co-folded poses), which could potentially greatly expand the dataset size at the cost of introducing additional uncertainties in terms of data errors. Another key component that should be considered is how to incorporate better property control. Unlike discrete architectures such as REINVENT [48] that provide direct access log-likelihoods, applying efficient RL to diffusion or flow-matching models is more challenging. Specifically, likelihood computation for these types of models is expensive due to having to propagate gradients through the entire time trajectory. This motivates more innovative model designs to facilitate practical, effective multi-dimensional steering.

## Figures and Tables

**Fig. 5:**
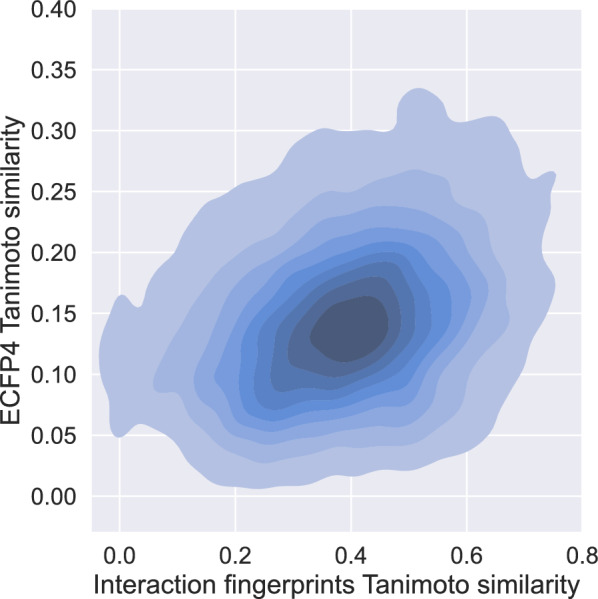
Two-dimensional distribution of scaffold and interaction similarities with native ligands

**Fig. 6:**
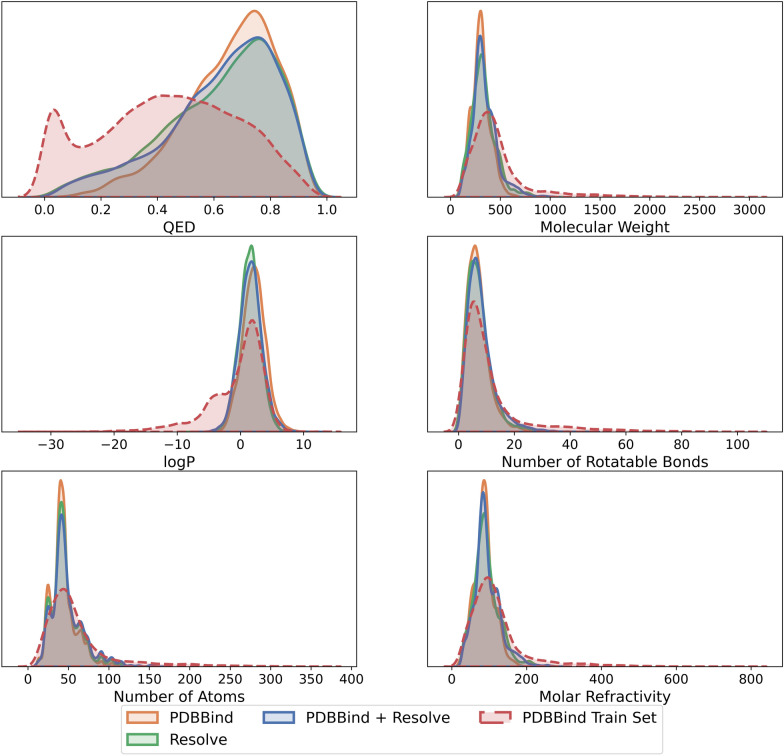
Overall chemical property distributions for molecules generated by three models

**Fig. 7:**
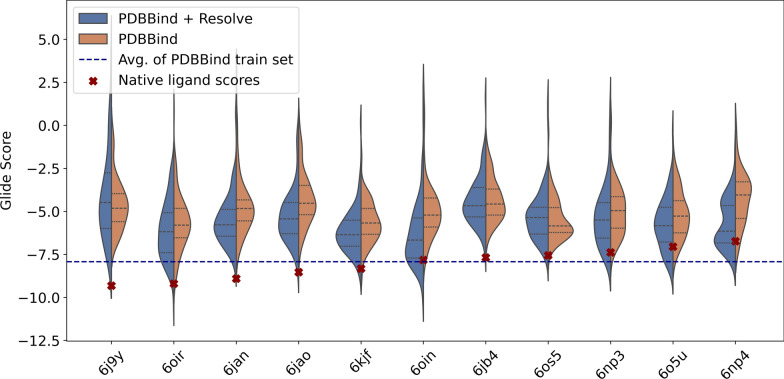
Glide score distributions for 11 representative protein targets. The native ligand’s Glide scores are labeled for each target. The blue line indicates the average Glide score for ligands in the PDBBind training set. Mean and standard deviation values are labeled in black lines for each system

**Fig. 8:**
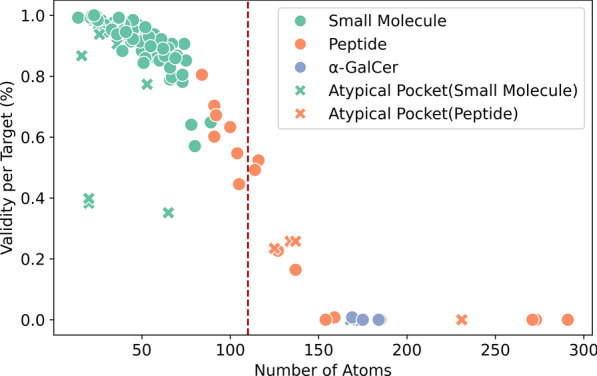
Validity ratio among different targets in PDBBind test set

**Fig. 9:**
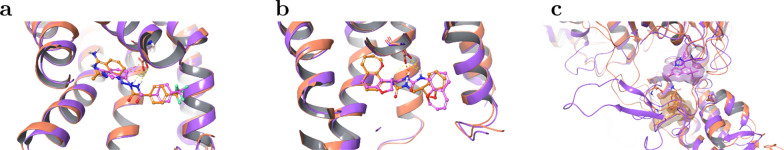
Comparison of Botlz-2 co-folded protein ligand complexes (purple) and the poses generated by our model (orange) for some of the protein ligand-pairs in Fig. 2f: 6IQL-BOTH (**a**), 6IQL-RES (**b**) and 6N9L-BOTH (**c**). Note the substantially different loop position and pocket placement between the two structures, rendering comparison challenging

## Data Availability

The raw ligand dataset GEOM-Drugs is available online at https://dataverse.harvard.edu/dataset.xhtml?persistentId=doi:10.7910/DVN/JNGTDF. The public protein-ligand complex dataset PDBBind (v.2020) can be downloaded at https://www.pdbbind-plus.org.cn/download.

## Appendix

### Appendix A: SPINDER dataset evaluation

**Table 7:** Molecule quality metrics for the SemlaFlow model

Method	QED	PBR (%)	BNR (%)	Valid (%)	# HB	S. Energy	Int.R (%)
SPINDER	$0.46 \pm 0.21$	49.6	$5.1 \pm 12.9$	94.10	$2.86 \pm 2.83$	$4.90 \pm 3.34$	$54.8 \pm 20.0$
SPINDER + Opt	$0.46 \pm 0.21$	76.7	$7.1 \pm 15.6$	94.10	$2.66 \pm 2.68$	$1.74 \pm 4.50$	$51.5 \pm 21.1$
PDBBind + Resolve	$0.59 \pm 0.18$	46.7	$6.8 \pm 15.4$	93.66	$2.37 \pm 2.24$	$4.21 \pm 3.80$	$53.6 \pm 18.8$
PDBBind + Resolve + Opt	$0.59 \pm 0.18$	75.1	$9.9 \pm 19.6$	93.66	$2.19 \pm 2.14$	$1.55 \pm 5.01$	$50.0 \pm 19.5$

*QED* quantitative estimate of drug-likeness, *PBR* PoseBuster pass ratio, *BNR* better-than-native ratio, Valid: validity ratio, *S. Energy* strain energy, *#HB.* hydrogen bond count, *Int.R* interaction recovery ratio

**Table 8:** Docking score evaluation for the SemlaFlow model

Method	$\text {VR} < 0$ (%)	$\text {GR} < 0$ (%)	VS	GS
SPINDER	87.36	44.49	$0.12 \pm 27.78$	$-3.93 \pm 3.88$
SPINDER + Opt	95.60	67.56	$-2.17 \pm 22.35$	$-3.10 \pm 6.21$
PDBBind + Resolve	79.70	43.54	$0.49 \pm 28.91$	$-3.45 \pm 5.14$
PDBBind + Resolve + Opt	92.59	72.94	$-2.57 \pm 21.11$	$-4.49 \pm 3.06$

*VR < 0* negative vina score ratio, *GR < 0* negative docking score ratio, *VS* vina score, *GS* glide score (mean docking score)

In this section, we utilized another curated protein-ligand dataset SPINDER to measure the generalizing ability of our model architecture. Different to experiments in "Effect of different datasets" section using PDBBind test set, here we use SPINDER test set for evaluation. Two models with the same architecture were compared before and after the optimization. One (“SPINDER") is trained from scratch on SPINDER training data, and the other (“PDBBind + Resolve", or “both") is the best-performing model from "Effect of different datasets" section. After optimization, the “both" model remained the good performance in terms of QED score, PoseBuster validity and strain energy (Table 7). Additionally, the “both" model achieved significant lower mean docking score (Table 8). These results collectively confirm the consistent superiority of SemlaFlow model trained on both PDBBind and Resolve datasets.

### Appendix B: Binding mode coherency

A successful model should be able to generate diverse structural motifs while keep consistent binding modes with the native ligand. To study the poses distribution, we analyzed the interaction and scaffold similarities of the molecules generated by our best-performing model against their native ligands (Fig. 5) using two descripitors: the ECFP4 fingerprint (1024 bit) Tanimoto similarity and the interaction fingerprint similarity [10]. The result demonstrated that the generated ligands successfully adopt diverse scaffolds while maintain similar interacting patterns, with interaction similarity scores centered around 40%.

### Appendix C: Chemical properties evaluation

To further validate the drug-likeness of generated molecules, we did a more rigorous analysis of their physicochemical properties shown (Fig. 6). All three models in "Effect of different datasets" section produced ligands that satisfied the Ghose filter [26]. Moreover, the distributions of the evaluated properties for the generated molecules were concentrated within ranges similar to those of the PDBBind training set.

### Appendix D: Glide score analysis

We took a deeper distributional assessment of the Glide scores for our generated molecules, focusing on 11 representative protein systems (Fig. 7). The specific targets were chosen to reflect the model’s performance across a diverse range of pockets, spanning from weak to strong native Glide scores. The results indicate that both the PDBBind and “both" models, as described in "Effect of different datasets" section, can generate adequate candidate ligands with acceptable docking score (<-5 kcal/mol) across various targets. However, the “both" model achieved lower overall Glide scores across most of the evaluated systems.

### Appendix E: Validity ratio for each target protein

The overall validity ratio is significantly influenced by extreme structural outliers within the test set. Figure 8 illustrates the per-system validity ratio of our best-performing model (“both") across the PDBBind test set. The heterogeneity of this test set introduces unique challenges for de novo generation. Specifically, the test set includes targets bound with non-standard native ligands, such as peptides and $\alpha$-GalCer analogues, as well as binding pockets of extreme sizes ($N_{atom} > 500$ or $< 120$). Generating molecules for these systems fall outside the intended application of the current generative model, resulting in suboptimal performance for these specific targets.

### Appendix F: Co-folding of generated ligands with Boltz-2

Co-folding methods such as AlphaFold 3 [38] and Boltz-2 [53] have attracted substantial interest. These models, building upon the original AlphaFold [38], take protein sequence and ligand SMILES (or other binder descriptors), and predict the structure of the protein-binder complex jointly. We stress that these models solve a fundamentally different task compared to those discussed here, namely pose (and potentially affinity) prediction vs. ligand design. To design new ligands with a co-folding method, it must be combined with an external ligand selection method such as virtual screening of a library or indeed a SMILES-based generative model [66]. These approaches typically require many thousands of inference calls against the large co-folding model, compared to direct generation afforded by pocket-conditioned generative models. While fair comparisons of the quality of results obtained by these different methods remain scarce, such a survey is unfortunately outside the scope of this work. However, we were interested to spot check the difference in pose quality obtained with our pocket-conditioned generative methods compared to a co-folding approach.

To this end, we co-folded the six ligands visualized in Fig. 2f with their respective targets using Boltz-2 (https://github.com/jwohlwend/boltz). We sampled 5 structures per complex using inference-time potentials, 3 recycling steps and 200 sampling steps. We selected the structures to analyze with the highest combined complex IPTM and confidence scores for each protein-ligand combination. Since Boltz-2 does not predict hydrogen atoms, we added them to the output poses using the same logic as in our hydrogen-free model ("Effect of incorporating hydrogen atoms during training" section).

An immediate issue with the comparison is that co-folding methods can and do place the ligand in a different pocket compared to the pocket generation method. For all PDB 6N9L structures, Boltz-2 consistently placed the ligand outside and above the original pocket, spatially separated from the original co-crystalzied binder, and also predicted a significantly different protein fold compared to the deposited structure which makes comparison of binding challenging (Fig. 9c). For PDB 6IQL, Boltz-2 consistently placed the ligand in the intended pocket, sometimes close to the pose we generate (Fig. 9a) and sometimes flipped the orientation inside the pocket (Fig. 9b).

We should point out that, without experimental verification, disambiguation of the correct binding mode is very challenging (if the proposed ligands binds at all). We can at least assess the geometric correctness of the ligand via strain energy and binding plausibility via the docking score and number of hydrogen bonds with the target. Considering 6IQL, we find that across the poses we studied, all metrics were generally comparable between the models, indicating similar geometric quality (Table 9).

**Table 9:** Molecule quality metrics for the SemlaFlow and Boltz-2 folded versions of the SemlaFlow ligands shown in Fig. 2 after local minimization

Target	Dataset	Boltz-2			SemlaFlow		
		S. Energy	GS	#HB	S. Energy	GS	#HB
6IQL	BOTH	0.69	$-7.66$	1	0.59	$-7.14$	1
	PDB	0.52	$-5.31$	1	0.73	$-5.65$	1
	RESOLVE	0.65	$-7.53$	1	0.33	$-6.61$	1
Average		0.62	$-6.83$	1.0	0.55	$-6.47$	1.0

*G.S* Glide Score, *S. Energy* strain energy, *# HB.* hydrogen bond count

## Code availability

The code will be made publicly available upon acceptance and a Github link will be added to the main text; it is currently accessible to reviewers during the revision period.

## References

[1] Adams K, Abeywardane K, Fromer J, Coley CW (2025) ShEPhERD: Diffusing shape, electrostatics, and pharmacophores for bioisosteric drug design. http://arxiv.org/abs/2411.04130

[2] Albergo MS, Vanden-Eijnden E (2023) Building normalizing flows with stochastic interpolants. arXiv:2209.15571

[3] Anderson AC (2003) The process of structure-based drug design. Chem Biol 10(9):787–797. 10.1016/j.chembiol.2003.09.002 14522049 10.1016/j.chembiol.2003.09.002

[4] Arús-Pous J, Blaschke T, Ulander S et al (2019) Exploring the GDB-13 chemical space using deep generative models. J Cheminform 11(1):20. 10.1186/s13321-019-0341-z 30868314 10.1186/s13321-019-0341-z PMC6419837

[5] Axelrod S, Gómez-Bombarelli R (2022) Geom, energy-annotated molecular conformations for property prediction and molecular generation. Sci Data 9(1):185. 10.1038/s41597-022-01288-4 35449137 10.1038/s41597-022-01288-4 PMC9023519

[6] Baillif B, Cole J, McCabe P et al (2024a) Benchmarking structure-based three-dimensional molecular generative models using GenBench3D: Ligand conformation quality matters. arXiv:2407.04424

[7] Baillif B, Cole J, McCabe P et al (2024b) Benchmarking structure-based three-dimensional molecular generative models using genbench3d: ligand conformation quality matters. arXiv:2407.04424

[8] Bickerton GR, Paolini GV, Besnard J et al (2012) Quantifying the chemical beauty of drugs. Nat Chem 4(2):90–98. 10.1038/nchem.1243 22270643 10.1038/nchem.1243 PMC3524573

[9] Bohacek RS, McMartin C, Guida WC (1996) The art and practice of structure-based drug design: a molecular modeling perspective. Med Res Rev 16(1):3–50 8788213 10.1002/(SICI)1098-1128(199601)16:1<3::AID-MED1>3.0.CO;2-6

[10] Bouysset C, Fiorucci S (2021) ProLIF: a library to encode molecular interactions as fingerprints. J Cheminform. 10.1186/s13321-021-00548-6 34563256 10.1186/s13321-021-00548-6 PMC8466659

[11] Buttenschoen M, Morris GM, Deane CM (2024) Posebusters: AI-based docking methods fail to generate physically valid poses or generalise to novel sequences. Chem Sci. 10.1039/D3SC04185A 38425520 10.1039/d3sc04185a PMC10901501

[12] Cai H, Shen C, Jian T et al (2024) CarsiDock: a deep learning paradigm for accurate protein-ligand docking and screening based on large-scale pre-training. Chem Sci 15(4):1449–1471. 10.1039/D3SC05552C 38274053 10.1039/d3sc05552c PMC10806797

[13] Campbell A, Benton J, De Bortoli V et al (2022) A continuous time framework for discrete denoising models. Adv Neural Inf Process Syst 35:28266–28279

[14] Campbell A, Yim J, Barzilay R et al (2024) Generative flows on discrete state-spaces: Enabling multimodal flows with applications to protein co-design. arXiv:2402.04997

[15] Chen RT, Rubanova Y, Bettencourt J (2018) Neural ordinary differential equations. Advances in neural information processing systems. p 31

[16] Corso G, Stärk H, Jing B (2023) DiffDock: Diffusion Steps, Twists, and Turns for Molecular Docking. arXiv:2210.01776

[17] Cremer J, Le T, Noé F (2024) Pilot: equivariant diffusion for pocket conditioned de novo ligand generation with multi-objective guidance via importance sampling. arXiv preprint arXiv:2405.14925 10.1039/d4sc03523b PMC11348832 39211741

[18] Cremer J, Irwin R, Tibo A (2025) Flowr: flow matching for structure-aware de novo, interaction-and fragment-based ligand generation. arXiv preprint arXiv:2504.10564 10.1038/s43588-026-00998-8 PMC13293864 42209794

[19] De Vivo M, Masetti M, Bottegoni G et al (2016) Role of molecular dynamics and related methods in drug discovery. J Med Chem 59(9):4035–4061. 10.1021/acs.jmedchem.5b01684 26807648 10.1021/acs.jmedchem.5b01684

[20] Dunn I, Koes DR (2024) Mixed continuous and categorical flow matching for 3d de novo molecule generation. arXiv:2404.19739 10.1039/d5dd00363f PMC13104864 42040512

[21] Durairaj J, Adeshina Y, Cao Z et al (2024) Plinder: the protein-ligand interactions dataset and evaluation resource. bioRxiv. 10.1101/2024.07.17.60395538979192

[22] Feng W, Wang L, Lin Z et al (2024) Generation of 3d molecules in pockets via a language model. Nat Mach Intell 6(1):62–73

[23] García-Ortegón M, Simm GNC, Tripp AJ et al (2022) DOCKSTRING: easy molecular docking yields better benchmarks for ligand design. J Chem Inf Model 62(15):3486–3502. 10.1021/acs.jcim.1c01334 35849793 10.1021/acs.jcim.1c01334 PMC9364321

[24] Gat I, Remez T, Shaul N (2024) Discrete flow matching. arXiv preprint arXiv:2407.15595

[25] Genheden S, Thakkar A, Chadimová V et al (2020) Aizynthfinder: a fast, robust and flexible open-source software for retrosynthetic planning. J Cheminform 12(1):70 33292482 10.1186/s13321-020-00472-1 PMC7672904

[26] Ghose AK, Viswanadhan VN, Wendoloski JJ (1999) A knowledge-based approach in designing combinatorial or medicinal chemistry libraries for drug discovery. 1. A qualitative and quantitative characterization of known drug databases. J Comb Chem 1(1):55–68. 10.1021/cc9800071 10746014 10.1021/cc9800071

[27] Grygorenko OO, Radchenko DS, Dziuba I et al (2020) Generating multibillion chemical space of readily accessible screening compounds. iScience 23(11):101681. 10.1016/j.isci.2020.101681 33145486 10.1016/j.isci.2020.101681 PMC7593547

[28] Guan J, Qian WW, Peng X et al (2023) 3d equivariant diffusion for target-aware molecule generation and affinity prediction. arXiv:2303.03543

[29] Guo J, Janet JP, Bauer MR et al (2021) DockStream: a docking wrapper to enhance de novo molecular design. J Cheminform 13(1):89. 10.1186/s13321-021-00563-7 34789335 10.1186/s13321-021-00563-7 PMC8596819

[30] Halgren TA, Murphy RB, Friesner RA et al (2004) Glide: a new approach for rapid, accurate docking and scoring. 2. Enrichment factors in database screening. J Med Chem 47(7):1750–1759. 10.1021/jm030644s 15027866 10.1021/jm030644s

[31] Harris C, Didi K, Jamasb AR et al (2023) Benchmarking generated poses: How rational is structure-based drug design with generative models? arXiv preprint arXiv:2308.07413

[32] Ho J, Jain A, Abbeel P (2020) Denoising diffusion probabilistic models. Adv Neural Inf Process Syst 33:6840–6851

[33] Hoogeboom E, Satorras VG, Vignac C (2022) Equivariant diffusion for molecule generation in 3d. International conference on machine learning. PMLR, p 8867–8887

[34] Hu Q, Sun C, He H et al (2025) Target-aware 3D molecular generation based on guided equivariant diffusion. Nat Commun 16(1):7928. 10.1038/s41467-025-63245-0 40854901 10.1038/s41467-025-63245-0 PMC12379259

[35] Huang L, Xu T, Yu Y et al (2024) A dual diffusion model enables 3D molecule generation and lead optimization based on target pockets. Nat Commun 15(1):2657. 10.1038/s41467-024-46569-1 38531837 10.1038/s41467-024-46569-1 PMC10965937

[36] Irwin R, Tibo A, Janet JP (2025) Semlaflow-efficient 3d molecular generation with latent attention and equivariant flow matching. International Conference on Artificial Intelligence and Statistics. PMLR, p 3772–3780

[37] Jeon W, Kim D (2020) Autonomous molecule generation using reinforcement learning and docking to develop potential novel inhibitors. Sci Rep 10(1):22104. 10.1038/s41598-020-78537-2 33328504 10.1038/s41598-020-78537-2 PMC7744578

[38] Jumper J, Evans R, Pritzel A (2021) Highly accurate protein structure prediction with alphafold. Nature 596(7873):583–589 34265844 10.1038/s41586-021-03819-2 PMC8371605

[39] Kim S, Chen J, Cheng T et al (2021) PubChem in 2021: new data content and improved web interfaces. Nucleic Acids Res 49(D1):D1388–D1395. 10.1093/nar/gkaa971 33151290 10.1093/nar/gkaa971 PMC7778930

[40] Koczor-Benda Z, Gilkes J, Bartucca F et al (2025) Structural bias in three-dimensional autoregressive generative machine learning of organic molecules. J Chem Inf Model 65(13):6644–6654. 10.1021/acs.jcim.5c00665 40556385 10.1021/acs.jcim.5c00665 PMC12264931

[41] Le T, Cremer J, Noé F (2023) Navigating the design space of equivariant diffusion-based generative models for de novo 3d molecule generation. arXiv

[42] Lee J, Zhung W, Seo J et al (2025) Bind: bond and interaction-generating diffusion model for multi-objective structure-based drug design. Adv Sci 12(35):e02702 10.1002/advs.202502702 PMC12463045 40642896

[43] Lipman Y, Chen R, Ben-Hamu H (2023) Flow matching for generative modeling. arXiv

[44] Liu H, Qin Y, Niu Z et al (2024) How good are current pocket-based 3D generative models?: The benchmark set and evaluation of protein pocket-based 3D molecular generative models. J Chem Inf Model 64(24):9260–9275. 10.1021/acs.jcim.4c01598 39629985 10.1021/acs.jcim.4c01598

[45] Liu M, Luo Y, Uchino K (2022) Generating 3d molecules for target protein binding. International Conference on Machine Learning. PMLR, p 13912–13924

[46] Liu X, Gong C, Qiang L (2023) Flow straight and fast: Learning to generate and transfer data with rectified flow. arXiv:2209.03003

[47] Liu Z, Li Y, Han L et al (2015) PDB-wide collection of binding data: current status of the PDBbind database. Bioinformatics 31(3):405–412. 10.1093/bioinformatics/btu626 25301850 10.1093/bioinformatics/btu626

[48] Loeffler HH, He J, Tibo A et al (2024) Reinvent 4: modern AI-driven generative molecule design. J Cheminform 16(1):20. 10.1186/s13321-024-00812-5 38383444 10.1186/s13321-024-00812-5 PMC10882833

[49] Luo S, Guan J, Ma J (2021) A 3d generative model for structure-based drug design. Advances in Neural Information Processing Systems. p 6229–6239

[50] Lyu J, Wang S, Balius TE et al (2019) Ultra-large library docking for discovering new chemotypes. Nature 566(7743):224–229. 10.1038/s41586-019-0917-9 30728502 10.1038/s41586-019-0917-9 PMC6383769

[51] Matricon P, Ranganathan A, Warnick E et al (2017) Fragment optimization for GPCRs by molecular dynamics free energy calculations: probing druggable subpockets of the A 2A adenosine receptor binding site. Sci Rep 7(1):6398. 10.1038/s41598-017-04905-0 28743961 10.1038/s41598-017-04905-0 PMC5526870

[52] McInnes L, Healy J, Melville J (2020) UMAP: uniform manifold approximation and projection for dimension reduction. arXiv. 10.48550/arXiv.1802.03426

[53] Passaro S, Corso G, Wohlwend J et al (2025) Boltz-2: towards accurate and efficient binding affinity prediction. bioRxiv. 10.1101/2025.06.14.65970741394589

[54] Peng X, Luo S, Guan J et al (2022) Pocket2Mol: Efficient molecular sampling based on 3D protein pockets. In: Proceedings of the 39th International Conference on Machine Learning, Proceedings of Machine Learning Research, vol 162. PMLR, p 17644–17655, arXiv:2205.07249

[55] Qu Y, Qiu K, Song Y (2024) Molcraft: Structure-based drug design in continuous parameter space. arxiv:2404.12141

[56] Ragoza M, Masuda T, Koes DR (2022) Generating 3D molecules conditional on receptor binding sites with deep generative models. Chem Sci 13(9):2701–2713. 10.1039/D1SC05976A 35356675 10.1039/d1sc05976a PMC8890264

[57] Rekesh A, Cretu M, Shevchuk D (2025) Syncogen: Synthesizable 3d molecule generation via joint reaction and coordinate modeling. arXiv preprint arXiv:2507.11818

[58] Röhrig UF, Mathieu-Bugnon M, Zoete V (2025) Comparative assessment of the utility of co-folding and docking for small-molecule drug design. bioRxiv. 10.64898/2025.12.09.693161

[59] Sanjrani N, Coupry DE, Pogány P et al (2025) Benchmarking 3D structure-based molecule generators. J Chem Inf Model 65(15):8006–8021. 10.1021/acs.jcim.5c01020 40711830 10.1021/acs.jcim.5c01020 PMC12344697

[60] Schneuing A, Du Y, Harris C (2023) Structure-based drug design with equivariant diffusion models. arXiv:2210.13695 10.1038/s43588-024-00737-x PMC11659159 39653846

[61] Schneuing A, Igashov I, Dobbelstein AW et al (2025) Multi-domain distribution learning for de novo drug design. arXiv:2508.17815

[62] Schrödinger LLC (2025) Schrödinger release 2025–2: maestro. Schrödinger, LLC, New York

[63] Shen T, Seo S, Irwin R (2025) Compositional flows for 3d molecule and synthesis pathway co-design In: Forty-second International Conference on Machine Learning

[64] Škrinjar P, Eberhardt J, Durairaj J (2025) Have protein-ligand co-folding methods moved beyond memorisation? bioRxiv. 10.1101/2025.02.03.636309

[65] Sohl-Dickstein J, Weiss E, Maheswaranathan N (2015) Deep unsupervised learning using nonequilibrium thermodynamics. International conference on machine learning. PMLR, p 2256–2265

[66] Thomas M, Bou A, Gómez-Tamayo JC et al (2025) Reinforce-ing chemical language models for drug discovery. J Chem Inf Model 65(23):12752–12763. 10.1021/acs.jcim.5c02053 41241844 10.1021/acs.jcim.5c02053 PMC12690592

[67] Tingle BI, Tang KG, Castanon M et al (2023) ZINC-22-A free multi-billion-scale database of tangible compounds for ligand discovery. J Chem Inf Model 63(4):1166–1176. 10.1021/acs.jcim.2c01253 36790087 10.1021/acs.jcim.2c01253 PMC9976280

[68] Trott O, Olson AJ (2010) AutoDock Vina: improving the speed and accuracy of docking with a new scoring function, efficient optimization, and multithreading. J Comput Chem 31(2):455–461. 10.1002/jcc.21334 19499576 10.1002/jcc.21334 PMC3041641

[69] Vignac C, Osman N, Toni L et al (2023) Midi: Mixed graph and 3d denoising diffusion for molecule generation. In: Machine Learning and Knowledge Discovery in Databases: Research Track - European Conference, ECML PKDD 2023, Turin, Italy, September 18-22, 2023, Proceedings, Part II, Lecture Notes in Computer Science, vol 14170. Springer, p 560–576, 10.1007/978-3-031-43415-0_33

[70] Wang L, Wu Y, Deng Y et al (2015) Accurate and reliable prediction of relative ligand binding potency in prospective drug discovery by way of a modern free-energy calculation protocol and force field. J Am Chem Soc 137(7):2695–2703. 10.1021/ja512751q 25625324 10.1021/ja512751q

[71] Wang R, Fang X, Lu Y et al (2005) The PDBbind database: methodologies and updates. J Med Chem 48(12):4111–4119 15943484 10.1021/jm048957q

[72] Yan J, Ye Z, Yang Z et al (2024) Multi-task bioassay pre-training for protein-ligand binding affinity prediction. Brief Bioinform 25(1):bbad451. 10.1093/bib/bbad451 10.1093/bib/bbad451 PMC10783875 38084920

[73] Yang B, Xiang C, Li T et al (2025) Structure-based generation of 3D small-molecule drugs: are we there yet? J Med Chem. 10.1021/acs.jmedchem.5c01706 41160754 10.1021/acs.jmedchem.5c01706 PMC13138256

[74] Zhang J, Mercado R, Engkvist O et al (2021) Comparative study of deep generative models on chemical space coverage. J Chem Inf Model 61(6):2572–2581. 10.1021/acs.jcim.0c01328 34015916 10.1021/acs.jcim.0c01328

[75] Zhang Z, Shen WX, Liu Q et al (2024) Efficient generation of protein pockets with Pocketgen. Nat Mach Intell. 10.1038/s42256-024-00920-940771998

[76] Zhang Z, Wang M, Liu Q (2024) Flexsbdd: structure-based drug design with flexible protein modeling. Adv Neural Inf Process Syst 37:53918–53944

[77] Zhou G, Gao Z, Ding Q (2022) Uni-Mol: a universal 3D molecular representation learning framework. 10.26434/chemrxiv-2022-jjm0j

[78] Zhung W, Kim H, Kim WY (2024) 3D molecular generative framework for interaction-guided drug design. Nat Commun 15(1):2688 38538598 10.1038/s41467-024-47011-2 PMC10973397

